# Some Records at Warrington

**Published:** 1919-02-15

**Authors:** 


### PUBLIC HEALTH REPORTS.

### Some Records at Warrington.

In his annual report for 1917, Dr. Joseph, the medical officer of health of the county borough of Warrington, gives an interesting survey of the sanitary conditions and the vital statistics of that industrial town. The vital statistics show that the health of Warrington during 1917 reached quite a high standard when consideration is given to the existing war conditions. The health standard of the borough for that year is indicated by the death-rate, which is 14.6, compared with 15.07 for the previous year, and by the fact that the number of cases of enteric fever and the death-rate from tuberculosis were the lowest on record, while the infant mortality had fallen from 96 to 92. The population for the year was estimated at 68,554 for the civilian death-rate; in 1914 the estimated population was 74,923. The birth-rate for 1917 is the lowest on record in the history of the town, being 22.3, compared with 25.1 for 1916. The zymotic death-rate was low except in the case of measles, which caused fifty deaths. There was no case of small-pox, and only one of cerebro-spinal fever, which proved fatal. Special attention was devoted during the year to the work of combating venereal diseases. The scheme which was brought into operation in March is a comprehensive one, and comprises free diagnosis, free treatment, the provision of salvarsan substitutes free of charge, and the education of the public. In the section dealing with maternity and child-welfare some interesting particulars are given regarding the causes of infant mortality. That the efforts of the Warrington health authorities to preserve and conserve child life are meeting with some measure of success is indicated by one significant fact—namely, the closer approximation between the death-rate among illegitimate children and the rate for legitimate children. The scheme for the prevention and treatment of tuberculosis is working well; during the year there were fewer deaths from this disease although more cases were notified, and the death-rate, which was 1.9 per 1,000, is the lowest on record for the borough.

